# Clinical study of Microendoscopic Discectomy + Fibrous Ring Suture Versus Microendoscopic Discectomy alone in the treatment of lumbar disc herniation in young and middle-aged patients

**DOI:** 10.12669/pjms.40.4.7935

**Published:** 2024

**Authors:** Hong-xun Cui, Yi-di Wang, Yong-hui Liu, Ma-long Guo

**Affiliations:** 1 Hong-xun Cui, Department of Orthopedics, Orthopedic Hospital of Henan Province, Luoyang 471000, Henan P.R. China; 2 Yi-di Wang, Department of Orthopedics, Henan University of Chinese Medicine, Zhengzhou, Henan, P.R. China; 3 Yong-hui Liu, Department of Orthopedics, Orthopedic Hospital of Henan Province, Luoyang 471000, Henan P.R. China; 4 Ma-long Guo, Department of Orthopedics, Orthopedic Hospital of Henan Province, Luoyang 471000, Henan P.R. China

**Keywords:** Microendoscopic, Lumbar disc herniation, Discectomy, Fibrous ring suture, Middle-aged and young adults

## Abstract

**Objective:**

To compare the clinical efficacy of microendoscopic discectomy + fibrous ring suture versus microendoscopic discectomy alone in the treatment of lumbar disc herniation (LDH) in young and middle-aged patients.

**Methods:**

A retrospective analysis was performed on the clinical data of 66 young and middle-aged patients with single-segment LDH diagnosed in Orthopedic Hospital of Henan Province from October 2019 to October 2022. All patients were divided into two groups: the microendoscopic discectomy + fibrous ring suture group and the microendoscopic discectomy alone group, with 33 cases in each group. The Visual Analogue Scale (VAS) and the Oswestry Disability Index (ODI) scores of the two groups were recorded before surgery and six and twelve months after surgery.

**Results:**

Both groups completed the surgery and postoperative follow-up successfully and showed no statistically significant differences in terms of incision length, duration of surgery, intraoperative blood loss and length of hospital stay (all P>0.05). VAS, ODI and JOA scores were significantly improved in both groups at 6 and 12 months after surgery compared with those before surgery (all P<0.05). The two groups were similar in terms of excellent and good rates of postoperative modified MacNab Evaluation Criteria, with no statistically significant differences. No serious complications were observed in the two groups during and after surgery.

**Conclusion:**

Both of the two surgical methods are effective in the treatment of LDH in young and middle-aged patients, and microendoscopic discectomy + fibrous ring suture in particular may be preferred because it results in significant improvement in patients’ VAS and ODI scores.

## INTRODUCTION

Lumbar disc herniation (LDH), a common degenerative disc disease in clinical orthopedics, is account for lumbar and leg pain. In terms of its pathological basis, the nucleus pulposus protrudes or extrudes from the ruptured fibrous ring, compressing the corresponding nerve roots. Intervertebral discs may protrude at all levels of the lumbar spine, with L4/5 and L5/S1 segments having the highest incidence. In clinical practice, patients with LDH are mainly characterized by lumbar and leg pain, mildly radiating pain in the lower limbs, restricted lumbar movement and abnormal sensation, and in severe cases, paraplegia.^1^

In China, young and middle-aged people are the mainstay of the workforce and a population vulnerable to LDH.^2^ The majority of young and middle-aged patients with LDH have a close bearing on long-term sitting, protracted standing or physical labor. Most patients with LDH are able to achieve symptomatic relief with conservative treatment, but some eventually require surgery due to recurrent or unremitting episodes of the disease. The rapid development of the percutaneous transforaminal endoscopic surgery (PETS) technique in recent years has made it a mainstream surgical procedure for the treatment of LDH.

Microendoscopic discectomy, while destroying the integrity of the annulus fibrosus, accelerates disc degeneration and makes it easier for the residual nucleus pulposus to re-protrude from the injured annulus fibrosus, leading to recurrence in patients with LDH after surgery. Supplemented by fibrous annulus suture, which promotes healing of the injured annulus fibrosus and effectively prevents re-protrusion of the residual nucleus pulposus, while slowing the rate of disc degeneration, with satisfactory recent clinical outcomes.^3^,^4^ In this study, a retrospective analysis was performed to compare the clinical efficacy of microendoscopic discectomy + fibrous ring suture versus microendoscopic discectomy alone in the treatment of young and middle-aged patients with LDH.

## METHODS

A retrospective study was used in this study. Sixty-six young and middle-aged LDH patients with informed consent who were admitted to the Orthopedic Hospital of Henan Province from October 2019 to October 2022 were selected as subjects. All patients were divided into two groups according to the surgical method: the microendoscopic discectomy + fibrous ring suture group (observation group) and the microendoscopic discectomy alone group (control group), with 33 cases in each group.

### Ethical Approval

The study was approved by the Institutional Ethics Committee of Orthopedic Hospital of Henan Province (No.: 2017-009-01; date: September 27, 2017), and written informed consent was obtained from all participants.

### Inclusion criteria:

- Age 18-50 years.
- Persistent or recurrent episodes of radicular pain.
- Clinical, CT and MRI confirmation of a single-segment LDH compressing a nerve root or dural sac in all cases.
- Clinical symptoms and signs consistent with the presentation of the herniated segment.
- Poor outcome after eight weeks of conservative treatment.
- Completed post-operative follow-up data and a follow-up period of over 12 months.

### Exclusion criteria:

- Patients with multi-segment disc herniation.
- Patients with non-discogenic or non-radicular pain.
- Patients who had undergone surgery or interventional therapy.
- Patients with psychiatric illness, severe cognitive impairment or communication difficulties.
- Patients with coronary heart disease, diabetes and other basic diseases;

Patients in the control group underwent microendoscopic discectomy alone. First, patients were placed under local anesthesia in a prone or lateral position, with routine disinfection and towel placement. The percutaneous puncture was performed approximately 12cm beside the midline of the spinal process. Subsequently, the puncture needle was inserted into the responsible segment with the help of a C’-arm machine, and a guide wire was inserted along the puncture needle. Then the needle was withdrawn, a surgical channel was established in the direction of the guide wire, and the herniated nucleus pulposus was removed microendoscopically.

Finally, the incision was rinsed with 0.9% sodium chloride solution, and radiofrequency haemostasis was applied. The working channel was withdrawn, the outer cuff was removed, and the incision was routinely sutured and dressed. In contrast, those in the observation group underwent microendoscopic discectomy + fibrous ring sutures. The nucleus pulposus was removed in the same way as in the control group. Care was taken to protect the fibrous ring during the incision and removal of the nucleus pulposus. Following the removal of the nucleus pulposus, the fibrous ring was sutured with a disposable fibrous ring suture, then the sutures were cut with special thread cutters. After checking the integrity of the fibrous ring, the ring was irrigated, radiofrequency haemostasis was performed, one negative pressure drain was placed, and then the incision was closed and the drainage tube was removed on the second postoperative day according to the drainage situation. All operations were performed by the same group of doctors.

### Postoperative management

Patients were placed in bed for one day after surgery and given medication for symptomatic treatment. On the 2^nd^ postoperative day, the patients were removed from the bed under the protection of a lumbar girth and straight leg-raising exercises were performed to prevent nerve root adhesion. Patients were discharged after 3-5 days with no fever and a stable condition. Free movement indoors was resumed one week after surgery and functional exercises for the lumbar and dorsal muscles were performed two weeks later. However, patients were still mainly confined to bed rest and were prohibited from lifting heavy objects for three months after surgery and from strenuous activities and heavy physical work for six months after surgery.

### Follow-up and observation indexes

Perioperative data, such as incision length, intraoperative blood loss, duration of surgery and length of hospital stay, were recorded for the two groups. The Visual Analogue Scale (VAS)^5^, the Oswestry Disability Index (ODI)^6^, the Japanese Orthopaedic Association (JOA)^7^ and the modified MacNab Evaluation Criteria^8^ were applied to evaluate the clinical outcomes of the two groups. At six and twelve months after surgery, patients were informed to review lumbar MRI on an outpatient basis. The size of the epidural indentation of the lumbar disc in the operated segment was measured on MRI before and after surgery, and the Pfirrmann grading of disc degeneration was performed at the same time.^9^,^10^

### Statistical analysis

All data in this study were statistically analyzed using SPSS20.0 software, and the measurement data were expressed as (*χ̅*;±*S*). Two independent sample *t* test was employed for comparison between the two groups, and paired t-test was utilized for comparison of pre-and-post-operative parameters within the groups. A 95% confidence interval was used. Besides, the c^2^ test was used for the incidence of complications between the two groups, with *P*<0.05 considered to be a statistically significant difference.

## RESULTS

In the observation group, there were 19 males and 14 females, aged 19-50 years, with a mean age of (38.48±8.18) years; of these, 20 had LDH in the L4/5 segment and 13 in the L5/S1 segment. While in the control group, there were 18 males and 15 females aged 19-49 years with a mean age of (37.45±9.51) years; of these, 18 had LDH in the L4/5 segment and 15 in the L5/S1 segment. No statistically significant differences were observed in preoperative indexes such as age, gender composition and lesion segments between the two groups (all *P*>0.05).

Both groups completed the surgery successfully without serious complications such as injury to major blood vessels, corresponding nerve roots and the dural sac during the operation. No statistically significant differences were observed in the incision length, intraoperative blood loss, duration of surgery and length of hospital stay between the two groups (all *P*>0.05), as shown in Table-I.

**Table-I:** Comparison of perioperative conditions between the two groups (*χ̅*±*S*).

Group	Incision length (cm)	intraoperative blood loss (ml)	Duration of surgery (min)	Length of hospital stay (d)
Control group	0.89±0.13	50.45±8.69	69.85±7.85	7.09±0.72
Observation group	0.87±0.12	46.67±8.45	71.97±7.17	6.91±0.98
*t* value	0.578	1.795	1.145	0.858
*P* value	0.565	0.077	0.256	0.394

The VAS, ODI and JOA scores of the two groups which are shown in Table-II. The VAS and ODI scores of the two groups were significantly decreased with the extension of time, with statistically significant differences at different time points (all *P*<0.05). JOA scores of the two groups were significantly increased with the extension of time, with statistically significant differences at different time points (all *P*<0.05). However, no statistically significant differences were observed in VAS, ODI and JOA scores between the two groups at the same time points (all *P*>0.05).

**Table-II:** Comparison of VAS, ODI and JOA scores between two groups at different times before and after surgery (*χ̅*±*S*).

Group	Time point	VAS score (points)	ODI score (points)	JOA score (points)
Control group	Before surgery	8.03±0.68	42.70±1.26	7.61±1.00
	6 months after surgery	2.27±0.45^a^	22.21±1.36^a^	20.18±1.51^a^
	12 months after surgery	0.45±0.51^a^	2.15±0.36^a^	25.18±1.10^a^
Observation group	Before surgery	7.94±0.70	42.55±1.37	8.03±0.77
	6 months after surgery	2.21±0.42^ab^	21.39±1.09^ab^	20.45±1.33^ab^
	12 months after surgery	0.61±.050^ab^	2.24±0.44^ab^	25.12±1.34^ab^

***Note:***

a P<0.05 compared with the group before surgery; ^b^ P>0.05 compared with the control group during the same period.

The excellent and good rates of postoperative modified MacNab Evaluation Criteria was 81.82% and 93.94%, respectively, with no statistically significant difference (*P*>0.05), as shown in Table-III Among them, three patients in the control group relapsed and underwent secondary surgery after failing conservative treatment, with satisfactory surgical results. Twelve months after surgery, the Pfirrmann grading of disc degeneration in the observation group was significantly improved compared with that before surgery, with a statistically significant difference (*P*<0.05). At six months and 12 months after surgery, the size of epidural indentation in both groups decreased significantly compared with that before surgery, with a statistically significant difference, and 12 months after surgery, the size of epidural indentation in the observation group was significantly smaller than that in the control group (all *P*<0.05), as shown in Table-IV.

**Table-III:** Comparison of MacNab excellent and good rate between the two groups 12 months after surgery [*n*, (%)].

Group	n	Excellent	Good	Medium	Poor	Excellent and good rate
Control group	33	19 (57.58)	8 (24.24)	5 (15.15)	1 (3.03)	27 (81.82)
Observation group	33	22 (66.67)	9 (27.27)	1 (3.03)	1 (3.03)	31 (93.94)
*c*^2^ value						2.276
*P* value						0.131

**Table-IV:** Comparison of Pfirrmann grading and epidural indentation between the two groups at different times before and after surgery (n,*χ̅*±*S*).

Group	Time point	Pfirrmann grading (n)		Epidural indentation (mm)

		Grade III	Grade IV	
Control group	Before surgery	13	20	7.76±0.56
	6 months after surgery	15	18	3.91±0.63^a^
	12 months after surgery	17	16	2.51±0.62^a^
Observation group	Before surgery	11	22	7.94±0.83
	6 months after surgery	16	17	3.61±0.61^a^
	12 months after surgery	20	13	1.52±0.51^ab^

***Note:***

a P<0.05 compared with the group before surgery; ^b^P>0.05 compared with the control group during the same period.

## DISCUSSION

It was shown in this study that there was no recurrence of LDH in the microendoscopic discectomy + fibrous ring suture group, whereas in the microendoscopic discectomy alone group, there was a recurrence of LDH in three patients who underwent secondary surgery after failing conservative treatment. Research showed a 6.5% recurrence rate in patients with single-segment disc herniation in the microendoscopic discectomy alone group, compared with none in the fibrous ring suture group.^11^

Research reported that none of the 36 LDH patients who underwent fibrous ring suture had recurrence at three years of follow-up, and regarded fibrous ring suture as a valuable technique for clinical application.^12^ More studies by Miller et al.^13^ suggested that fibrous ring suture was effective in avoiding the re-protrusion of the nucleus pulposus from the rupture. Theoretically, the residual nucleus pulposus can be brought into contact with nerve roots and dural membrane after suture at the annulus fibrosus rupture, reducing postoperative scar formation. In the follow-up of this study, patients in the microendoscopic discectomy + fibrous ring suture group had almost no epidural indentation compared with those in the microendoscopic discectomy alone group, indirectly demonstrating the effectiveness of fibrous ring suture in reducing postoperative scar formation.

In the distant future, patients with recurrent LDH may have difficulty separating the dural sac from the surrounding tissue scar during surgery because of their obvious adhesion. Forced separation of the two may cause neural tube damage and tearing of the dura, resulting in increased surgical difficulty. Microendoscopic discectomy is superior to open surgical procedures due to the following advantages:

- It is a minimally invasive procedure with small damage to the posterior soft tissues, ligaments and muscles of the lumbar spine, which neither destroys the structure of the spine itself nor affects spinal stability.
- It is performed with a small incision, mostly within 1cm, and a small postoperative scar, compared to the large scar caused by traditional open surgical procedures.
- It results in less blood loss, short bed time and quick recovery after surgery. Internationally, in 1991, Yeung et al.^14^ pioneered the use of the YESS technique via the posterior lateral approach for the treatment of LDH in 1991; Literature has shown used the TESSYS technique for LDH around 2001. All these methods have achieved satisfactory clinical outcomes. Nowadays, microendoscopic discectomy has become a mainstream minimally invasive method for the treatment of LDH.^15^,^16^

The fibrous ring of the lumbar disc, a key structure for maintaining the stability of the nucleus pulposus, plays an extremely important role in the function of the lumbar disc. Given its poor blood supply and poor ability to repair itself, the fibrous ring of the lumbar disc can only take scar healing as the main curative effect, and its strength after healing is worse than that of normal ones. However, microendoscopic discectomy alone cannot repair the fibrous ring fissure, resulting in the persistence of the fissure, whereby the residual nucleus tends to protrude from the ruptured fibrous ring again, leading to the recurrence of LDH.^17^ Literature has shown a 10%-15% recurrence rate of LDH after microendoscopic discectomy.^18^ It was also reported that 15% of LDH patients underwent secondary surgery within eight years after simple discectomy, among which 55% had secondary surgery within two years after surgery, and up to 62% had secondary surgery due to postoperative recurrence.^19^ At present, no significant progress has been made in the repair and regeneration of fibrous ring. Therefore, clinicians usually do not treat the fibrous ring intraoperatively. Instead, they remove as much disc tissue as possible to avoid recurrence, which accelerates disc height loss and spinal instability.

In this study, postoperative VAS, ODI and JOA scores were significantly reduced in both groups compared to the preoperative period, with similar good and good rates of surgery as well as significant short-term clinical outcomes. However, the Pfirrmann grading of the degree of disc degeneration in the operated segments showed a significant difference in the preoperative and postoperative degeneration of the patients in the microendoscopic discectomy + fibrous ring suture group, indicating that there was “rehydration” of the lumbar disc in this group. This kind of “rehydration” is of great importance in patients with LDH, slowing down the degeneration of the lumbar disc and serving as a reminder of good long-term clinical outcomes. However, long-term clinical follow-up and studies are still needed to confirm this.

### Limitations of the study

It includes the inability to restore the tissue integrity of the fibrous ring, which need to be further investigated by clinical practitioners. Moreover, long-term follow-up is required for the long-term efficacy of this technique because of the short follow-up time in this study.

## CONCLUSION

Both surgical methods of microendoscopic discectomy + fibrous ring suture and microendoscopic discectomy alone are effective in the treatment of lumbar disc herniation in young and middle-aged patients, which are minimally invasive spinal surgery techniques worthy of clinical promotion. Despite the similar early clinical results of the two procedures, microendoscopic discectomy + fibrous ring suture may promote fibrous ring repair and theoretically has a lower rate of postoperative LDH recurrence.

### Authors’ Contributions:

**HC** and **MG:** Carried out the studies, participated in collecting data, drafted the manuscript, are responsible and accountable for the accuracy and integrity of the work. **YW:** Performed the statistical analysis and participated in its design. **YL:** Participated in acquisition, analysis, or interpretation of data and draft the manuscript. All authors read and approved the final manuscript.
